# Investigating Macrolactone Formation by Thioesterase Domains through Incorporation of an Unnatural Amino Acid

**DOI:** 10.1063/4.0000900

**Published:** 2025-10-27

**Authors:** Vishakha Choudhary, Tyler M. McCullough, Janet L. Smith

**Affiliations:** University of Michigan, Ann Arbor, MI, USA

## Abstract

Vishakha Choudhary, Tyler M. McCullough, David L. Akey, Meredith A. Skiba, Steffen M. Bernard, Jeffrey D. Kittendorf, Jennifer J. Schmidt, David H. Sherman and Janet L. Smith

Life Sciences Institute, Department of Biological Chemistry, Department of Medicinal Chemistry, Department of Chemistry, Department of Microbiology & Immunology, University of Michigan, Ann Arbor, MI

Macrolide antibiotics such as pikromycin and erythromycin are therapeutically relevant polyketide natural products. Formation of the macrolactone core relies on cyclization by type I thioesterase (TE) domains. TEs contain a catalytic triad composed of a Ser or Cys nucleophile, a His and an Asp. Like serine proteases, TEs form an acyl-enzyme intermediate of the substrate at the catalytic Ser or Cys. The intermediate is resolved by either nucleophilic attack of a substrate hydroxyl to form a macrolactone or hydrolysis to a linear product. The biochemical understanding of how TE domains control macrolactone formation vs. linear product release remains unclear. To understand the control of cyclization vs. hydrolysis, we aim to visualize acyl-enzyme intermediates, but this is challenging due to their lability. We used a strategy that replaces the catalytic Ser or Cys with the unnatural amino acid 2,3-diaminopropionic acid (DAP)^1^. DAP is a Ser/Cys isostere with an amino functionality that can form a stable amide linkage to substrates. DAP incorporation enables visualization of a diverse array of irreversibly linked natural and unnatural polyketide substrates in TE active sites. We incorporated DAP in the active site of the pikromycin PikAIV TE and the erythromycin DEBSIII TE, used intact protein mass spectrometry to detect the trapped intermediates and to investigate substrate scope, and solved a crystal structure for PikAIV TE-DAP adduct of the natural heptaketide substrate^2^. The biochemical and structural investigation of substrate engagement with the active site provides new insights into substrate tolerance and stereoselectivity in TE domains. This work lays a foundation for understanding diversification of substrates as well as the synthesis of novel macrolactone products in TE domains, which are valuable engineering targets.
